# Antigen presentation of post‐translationally modified peptides in major histocompatibility complexes

**DOI:** 10.1111/imcb.12839

**Published:** 2024-11-28

**Authors:** Alexine S de Wit, Frans Bianchi, Geert van den Bogaart

**Affiliations:** ^1^ Department of Molecular Immunology, Groningen Biomolecular Sciences and Biotechnology Institute University of Groningen Groningen The Netherlands

**Keywords:** Adaptive immune system, antigen presentation, major histocompatibility complex, post‐translational modification, T cells

## Abstract

T cells of the adaptive immune system recognize pathogens and malignantly transformed cells through a process called antigen presentation. During this process, peptides are displayed on major histocompatibility complex (MHC) class I and II molecules. Self‐reactive T cells are typically removed or suppressed during T‐cell development and through peripheral tolerance mechanisms, ensuring that only T cells recognizing peptides that are either absent or present in low abundance under normal conditions remain. This selective process allows T cells to respond to peptides derived from foreign proteins while ignoring those from self‐proteins. However, T cells can also respond to peptides derived from proteins that have undergone post‐translational modifications (PTMs). Over 200 different PTMs have been described, and while they are essential for protein function, localization and stability, their dysregulation is often associated with disease conditions. PTMs can affect the proteolytic processing of proteins and prevent MHC binding, thereby changing the repertoire of peptides presented on MHC molecules. However, it is also increasingly evident that many peptides presented on MHC molecules carry PTMs, which can alter their immunogenicity. As a result, the presentation of post‐translationally modified peptides by MHC molecules plays a significant role in various diseases, as well as autoimmune disorders and allergies. This review will provide an overview of the impact of PTMs on antigen presentation and their implications for immune recognition and disease.

## Graphical abstract

T cells recognize pathogens and malignantly transformed cells through antigen presentation on major histocompatibility complex molecules. Post‐translational modifications (PTMs) of proteins can alter the peptides presented, influencing immune recognition and disease. This review examines how PTMs affect antigen presentation and their role in immune responses and various diseases.

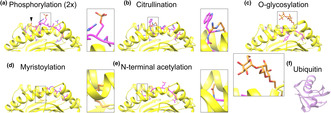

## INTRODUCTION

Major histocompatibility complex (MHC) class I and II molecules play vital roles in the immune system by presenting antigenic peptides to T cells, initiating immune responses against pathogens and malignantly transformed cells. MHC class I presentation mainly involves intracellularly produced proteins, which are degraded into peptides by the proteasome and loaded onto MHC class I molecules within the endoplasmic reticulum (ER). These peptide–MHC class I complexes are then displayed on the cell surface for recognition by the T‐cell receptor (TCR) of CD8^+^ cytolytic T cells, leading to the elimination of infected or cancer cells. By contrast, the main source of MHC class II presented peptides are extracellular pathogens and other antigens. Following their engulfment by professional antigen‐presenting cells, including macrophages, B cells and dendritic cells, these are processed into peptides within endosomes/phagosomes and loaded onto MHC class II molecules. Peptide‐loaded MHC class II complexes are then presented on the cell surface for recognition by CD4^+^ helper T cells, which activate immune responses by releasing cytokines and providing signals to other immune cells.

To ensure self‐tolerance and prevent autoimmunity, developing T cells undergo the process of central tolerance in the thymus where negative selection of self‐reactive TCRs takes place. Precursor T cells that express TCRs that strongly bind to self‐antigens presented by MHC molecules on thymic epithelial cells (TECs) receive apoptotic signals, leading to their elimination through programmed cell death. TECs have the unique ability to express a wide array of self‐proteins through a process known as promiscuous gene expression, whereby TECs transcriptionally activate genes typically restricted to specific tissues, called tissue‐restricted antigens. The autoimmune regulator (AIRE) protein plays a central role in this process by promoting the expression of tissue‐restricted antigens in TECs. AIRE accomplishes this by binding to chromatin within the nucleus of TECs and facilitating the transcription of these genes, reflecting the protein repertoire found throughout the body. This process ensures the deletion of autoreactive T cells from the T‐cell repertoire, minimizing the risk of autoimmune reactions. In addition, regulatory T cells (T_regs_) may develop from a subset of autoreactive thymocytes, serving to suppress the activation and function of remaining autoreactive T cells in the periphery, thus contributing to tolerance and preventing autoimmune diseases.

**Figure 1:**
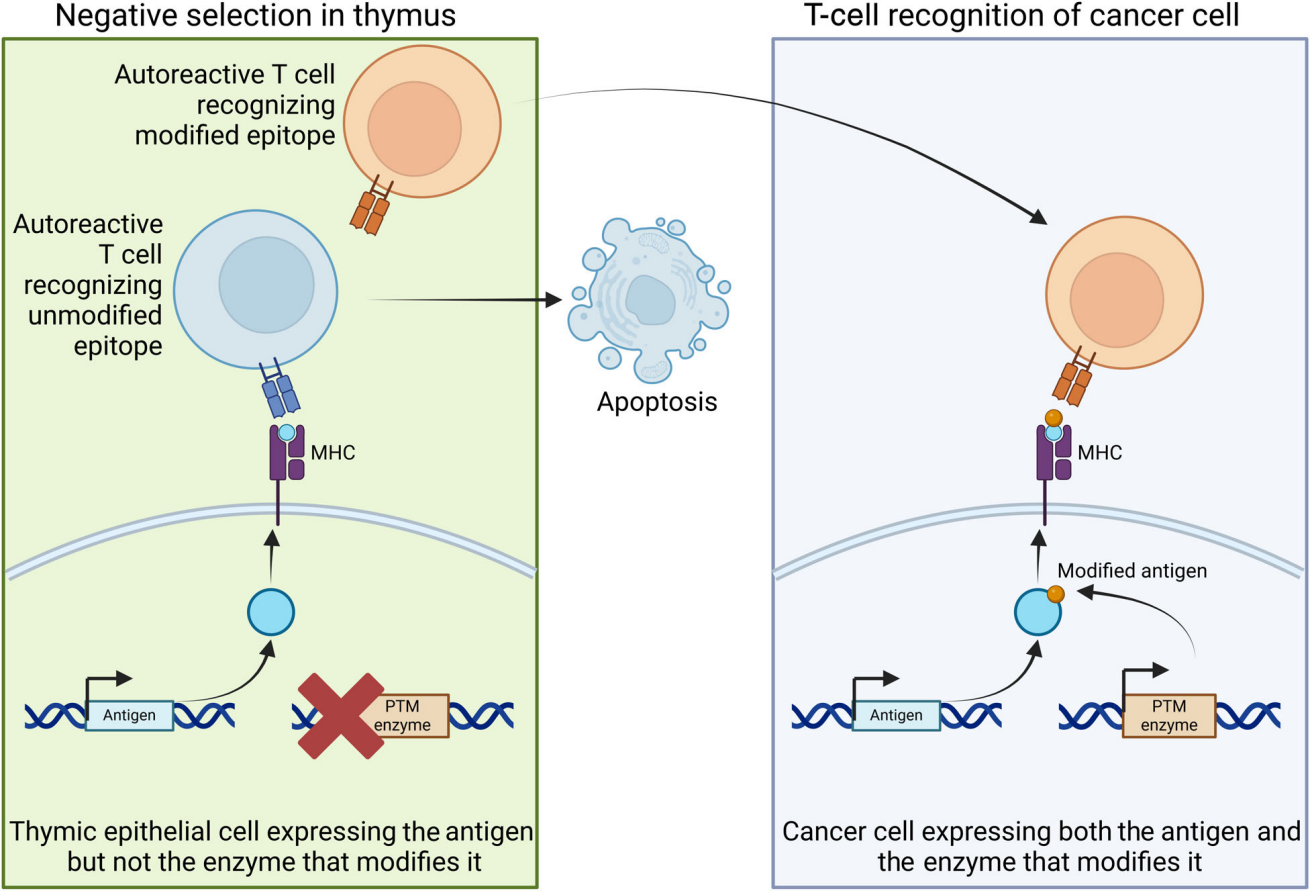
Model of the role of post‐translational modifications (PTMs) in T‐cell cancer recognition. Thymic epithelial cells express tissue‐restricted antigens, but not the enzymes responsible for their PTM such as kinases and glycosyl transferases. Therefore, negative selection only removes T cells recognizing the unmodified major histocompatibility complex (MHC) epitopes. As cancer cells express both the antigen and the enzymes responsible for PTMs, they can be recognized by T cells that recognize the modified epitopes. This figure was created with Biorender.com.

As TECs express random proteins, but not necessarily the interacting proteins and signaling mechanisms, these proteins can be expected to lack post‐translational modifications (PTMs). For example, T‐cell autoimmunity to collagen II is biased toward a glycosylated immunodominant epitope in rheumatoid arthritis (RA),^1^, ^2^ implying thymic T‐cell tolerance is strictly for nonglycosylated collagen II. In line with this, collagen II modified with hydroxylysine glycosylation was shown to be excluded from negative selection by TECs, despite that peptides carrying this modification could induce autoreactive T cells in a mouse model of autoimmunity.^3^ Instead, it was shown that thymic tolerance to the post‐translationally modified epitope depended on the delivery of the antigen to the thymus, likely by dendritic cells.^3^ Thus, negative selection can incompletely filter out post‐translationally modified proteins (Figure 1).

Post‐translational modifications are well understood to affect MHC recognition in two ways. First, PTMs can affect the generation of MHC epitopes by altering protein cleavage, for instance, through masking of protease cleavage sites, thereby potentially altering the MHC peptide repertoire.^4^ Furthermore, the presence of isoaspartate in the model antigen pigeon cytochrome c alters its cleavage by the lysosomal protease cathepsin D, and thereby facilitates T‐cell recognition of the altered epitopes.^5^ Similarly, glycosylation of the human immunodeficiency virus (HIV) envelope glycoprotein gp120 affects proteolytic processing and thereby MHC‐II presentation of nearby epitopes.^6^ A special case of altered proteolytic processing is MHC class I epitopes that are generated by the splicing of discontinuous peptide segments by the proteasome,^7^, ^8^ as reviewed elsewhere.^9^

Second, the PTM might prevent or facilitate the binding to the MHC. In fact, numerous post‐translationally modified peptides have been identified directly on both MHC class I and II through peptide elution studies using mass spectrometry (MS).^10^, ^11^, ^12^, ^13^, ^14^ However, the identification of PTMs in MS data is challenging because of the vast number of over 200 different known PTMs that significantly increase the search space for peak annotation. Recent improvements in the hardware and software for analysis of MS data enabled the identification of MHC‐bound peptides carrying many different PTMs.^15^, ^16^, ^17^, ^18^, ^19^ For example, a potent strategy for identification of post‐translationally modified peptides is to first find approximate matches from the tandem mass spectrometry spectrum in a sequence database and then select the best match by refining the annotation by assigning the mass differences to PTMs.^20^

Whereas some PTMs are small and might not significantly influence MHC presentation, others are quite large, raising questions about their influence on the interaction between peptide–MHC complexes and TCR, as well as how these modified peptides are processed. As many PTMs are dysregulated in pathological conditions, such as inflammation and cancer, the repertoire of MHC‐presented peptides often differs between healthy and diseased states. This variation plays a key role in how the immune system identifies infected or malignantly transformed cells but also contributes to autoimmune diseases and allergies.^21^ These topics will be the focus of this review.

## EVIDENCE OF PTMS IN MHC CLASS I AND CLASS II

Tables 1, 2, 3, 4, 5, 6, 7 provide an overview of the different PTMs that have been reported on MHC‐presented peptides and their association with pathological conditions. Although not exhaustive, these tables offer a comprehensive representation of the types of modification and their targets. Some of these modifications involve the addition of small modifications to the epitope, such as phosphorylation, which has been observed for peptides presented in both MHC class I^22^, ^23^, ^24^, ^25^, ^26^, ^27^ and MHC class II^24^, ^27^, ^28^ or acetylation, a modification shown to be important in the immunogenicity of a peptide derived from tumor protein p53.^29^ By contrast, large PTMs such as lipidation or glycosylation involve the addition of more complex molecules to proteins. One form of lipidation is N‐myristoylation, where a saturated C14 fatty acid (myristic acid) is linked to an N‐terminal glycine, anchoring the protein to the cell membrane after the removal of the starting methionine.^11^ Glycosylation, the enzymatic addition of glycans to the N and O groups of specific amino acid residues, was once thought to be incompatible with MHC presentation because of the size of the modification.

**Table 1:** Glycosylation and asparagine deamidation^a^.

PTM	Protein	MHC‐I or ‐II	Allotype	Disease association	Reference
O‐linked β‐GlcNAc	Various	MHC‐I	HLA‐B*07:02	Leukemia	48
	Various	MHC‐I			52
	Various	MHC‐I	HLA‐B*07:02		53
	Various	MHC‐I	HLA‐B*07:02 and ‐A*03:01		119
	Hemoglobin	MHC‐II	I‐E^k^		102
	Sendai virus nucleoprotein	MHC‐I	H‐2K^b^ and H‐2D^b^	Infections	120, 121, 122
N,O‐glycosylation	Various	MHC‐II	HLA‐DR	Melanoma	18
N‐glycosylation	CD53	MHC‐II	HLA‐DR4		123
	CII	MHC‐II	I‐A^q^	Rheumatoid arthritis	34, 111, 112
	Model antigen OVA	MHC‐II	I‐A^d^		30
	Bee venom PLA2	MHC‐II	HLA‐DR	Bee allergy	80
	Various	MHC‐I	H‐2^b^	Cancer	124
	Various	MHC‐II			19
O‐glycosylation	Model antigen HEL	MHC‐II	I‐A^k^		31
	Vesicular stomatitis virus nucleoprotein	MHC‐I	H2‐K^b^	Infections	59
	MUC1	MHC‐II	I‐A^b^ and I‐A^d^	Adenocarcinomas	36, 37, 38
	MUC1 and Sendai virus nucleoprotein	MHC‐I	H2‐K^b^	Adenocarcinomas	35
N deamidation	HCVE1	MHC‐I	Patr‐B*1601	Hepatitis C in chimpanzee	103
	Model antigen HEL	MHC‐II	I‐A^k^		104
	Various	MHC‐I			16
	Various	MHC‐I		Leukemia and melanoma	17
	TYR	MHC‐I	HLA‐A*02:01	Melanoma	40, 41, 45
	Choriomeningitis virus GP1	MHC‐I	H2‐D^b^	Infections	46
	HIV‐1 protein env	MHC‐I	HLA‐Cw*08 and ‐B7	Infections	47
	Model antigen	MHC‐I	H2‐K^b^		42
	Various	MHC‐I			44
	Various	MHC‐I		Cancer	15
Artificial sugar conjugation	Vesicular stomatitis virus nucleoprotein	MHC‐I	H2‐K^b^		125
Monoglycosylation (galactose)	Guinea pig MBP	MHC‐II	Rat RT1.BL		79
O‐linked beta‐d‐galactopyranose and disaccharide	CII	MHC‐II	H‐2q and HLA‐DR4	Rheumatoid arthritis	1, 2, 32, 34
N‐terminal glycosylation	Model antigen HEL	MHC‐II	I‐A^k^		126
Hydroxylysine glycosylation	CII	MHC‐II	I‐A^q^	Rheumatoid arthritis	3, 33, 127

HEL, hen egg lysozyme; HIV, human immunodeficiency virus; MHC, major histocompatibility complex; PTM, post‐translational modification.

^a^ Although deamidation can occur as an experimental artifact, deglycosylation of asparagine residue catalyzed by the NGLY1 enzyme also leads to deamidation.

**Table 2:** Phosphorylation.

Protein	MHC‐I or ‐II	Allotype	Disease association	Reference
CDC25b	MHC‐I	HLA‐A2	Cancer	22, 128
IRS2 and BCAR3		HLA‐A*02:01	Cancer	22, 106, 110, 128
TRAP1		HLA‐A2	Cancer	129
Synthetic antigen RR10		HLA‐B27		116
Various		HLA‐A*02:01; HLA‐B*07:02	Leukemia	109
		HLA‐B40, ‐B27, ‐B39 and ‐B7		26
		HLA‐A2, A24, B7 and B34		24
		HLA‐B40		23
		HLA‐A2		51, 57, 58
				16
			Leukemia and melanoma	17
		HLA‐A1, ‐A*02:01, ‐A3, ‐A*68:02, ‐B7, ‐B8, ‐B27 and HLA‐B35		22, 25
		HLA‐A2 and ‐B35	Leukemia	109
			Cancer	15
VIME	MHC‐II	HLA‐DR14 and ‐DR53	Colorectal cancer	130
TP53		HLA‐DR1 and ‐DR9	Head and neck squamous cell carcinoma	131
BRAF and MLANA		HLA‐DR1	Melanoma and leukemia	28, 55
Various			Melanoma and Epstein–Barr virus–transformed B lymphoblastoma	28
		HLA‐DRB1		24

MHC, major histocompatibility complex; PTM, post‐translational modification.

**Table 3:** Cysteine modifications.

PTM	Protein	MHC‐I or ‐II	Allotype	Disease association	Reference
Cysteinylation	KDM5D	MHC‐I	HLA‐A*02:01	Organ rejection	99
	USP9Y	MHC‐I	HLA‐A*01:01	Organ rejection	100
	Various	MHC‐I			16
	Influenza nuclear protein	MHC‐I	H2‐K^d^	Influenza	69
	Various	MHC‐I		Cancer	15
Disulfide bridge formation	INS	MHC‐II	HLA‐DR4	Type I diabetes	132
	Various	MHC‐II	HLA‐DR	Melanoma	18
Drug conjugation	RASK	MHC‐I	HLA‐A*02:01 and A*03:01	Lung and colon cancer	107
Carbamidomethylation	Various	MHC‐I		Cancer	15
Oxidation	Various	MHC‐I		Cancer	15
Methylation	Various	MHC‐I		Cancer	15

MHC, major histocompatibility complex; PTM, post‐translational modification.

However, it is now clear that glycosylated peptides can be presented on MHC without the removal of the saccharide moiety. For example, an MHC class II peptide elution study identified a large number of glycopeptides carrying 17 different glycosylation moieties in melanoma and Epstein–Barr virus–transformed cell lines^18^ and a recent reanalysis of eight published MHC human peptide elution proteomics data sets revealed over 3400 N‐glycosylated epitopes.^19^ Moreover, T cells that specifically recognize glycosylated epitopes presented on MHC class II have been identified in mice immunized with a glycopeptide.^30^ In another experiment, CD8^+^ T‐cell activation was abolished in mice immunized with the model antigen hen egg lysozyme when the O‐linked disaccharide was removed or replaced with another sugar.^31^ Other studies also found that MHC presented glycosylated peptides with the sugar chains still attached and T‐cell responses toward glycosylated autoantigens have been associated with RA^3^, ^32^, ^33^ and cancer.^34^ In humans, T cells have been identified that specifically recognize MHC class I‐ and class II‐presented peptides with incomplete glycosylation sites from the human mucin–type protein MUC1.^35^, ^36^, ^37^, ^38^ These findings demonstrate that glycosylated peptides can indeed be presented on both MHC class I and II.

In other cases, the modification involves the conversion of a residue into another molecule. A well‐known example is citrullination (also known as deimination), where arginine is irreversibly converted into citrulline by peptidylarginine deiminase. It has been demonstrated that citrullinated peptides are expressed on tumors, and specific T cell clones recognizing these citrullinated peptides have been shown to elicit antitumor responses.^39^ Another type of conversion is deamidation, where glycosylated asparagine is converted into aspartic acid following deglycosylation by N‐glycanase 1 (NGLY1) in the cytosol, before deglycosylated peptides are imported into the ER by the transporter associated with antigen presentation (TAP).^12^, ^40^, ^41^, ^42^ This altered residue is frequently observed in peptide elution studies.^43^ However, deamidation can also occur as an experimental artifact during sample processing, complicating the interpretation of detected modifications. Interestingly, an analysis of MS data revealed that almost half of the deamidated residues were located in the N‐glycosylation motif N–X(P)–S/T, suggesting that deglycosylation of the peptides occurred before the MHC presentation.^17^ Moreover, it was found that inhibition of NGLY1 reduced the number of deamidated peptides in a peptide elution study^44^ and that HLA‐A*01:01 only presented deamidated peptides, but not their nonmodified counterparts.^15^ Here, the deamidate site was preferentially located at position 3 of the peptide epitope, which makes sense as asparagine is converted to aspartate that appears in the HLA‐A*01:01 binding motif at position 3.^15^ Finally, CD8^+^ T cells have been identified that specifically recognize deamidated epitopes from tyrosinase in melanoma,^45^ choriomeningitis virus (lymphocytic choriomeningitis virus) GP1 glycoprotein^46^ and HIV‐1 viral envelope protein.^47^ These findings show that deglycosylation can occur before MHC loading.

In pathological conditions, altered PTMs are often the result of changed activities of enzymes, such as dysregulated kinase and phosphatase activities in cancer and inflammation, or incomplete glycosylation in cancer.^48^ However, PTMs can also be the result of spontaneous nonenzymatic PTMs. For instance, inflammation results in the production of nitric oxide and reactive oxygen species that oxidatively modify tyrosines, cysteines, methionines, histidines, prolines and tryptophans, and peptides with oxidized tyrosines have been found on mouse MHC class II.^49^, ^50^

## ACCOMMODATION OF POST‐TRANSLATIONALLY MODIFIED PEPTIDES ON MHC AND INTERACTION WITH TCR

Intriguingly, some PTMs are very large, such as glycosylation discussed above. The addition of a single saccharide will add considerably to the size of the epitope, because the average molecular weight of a monosaccharide is about 180 Da, which is larger than the 110 Da of the average amino acid. Other large PTMs can also be presented on MHC. For example, the MHC class II presentation of peptides that are linked by a disulfide bond to another peptide has been reported.^18^ Even more extreme examples of MHC epitopes carrying large PTMs are FAT10 conjugation, SUMOylation and ubiquitination, where the peptide is bound to at least one copy of an 18.5‐, 11‐ and 8.5‐kDa protein, respectively. Surprisingly, these modifications have been identified in a peptide elution study on MHC class I,^15^ although it is unclear how they affect T‐cell recognition.

For MHC class II presentation of epitopes carrying PTMs, the HLA‐DM peptide loading complex needs to accommodate these (sometimes large) modifications. For MHC class I, peptides can spontaneously be loaded in the peptide binding groove following exchange with other peptides. However, cytosolic peptides carrying PTMs still need to be imported into the ER lumen by TAP. Indeed, for phosphorylated MHC class I epitopes this has been shown to be the case.^51^ In addition, larger modifications seem to be imported in the ER as peptides linked to the cytosolic PTM O‐β‐linked N‐acetylglucosamine (O‐GlcNAc) are frequently found on MHC class I,^52^ with MS revealing that most O‐GlcNAcylated peptides are derived from nuclear proteins.^53^ For PTMs involving linkage to proteins, such as FAT10, SUMO and ubiquitin, it is a mystery how they would enter the ER. Possibly, MHC class I presentation of such large modifications does not involve TAP at all but the peptides might reach the lumen of the ER in a different manner (e.g. through some autophagy‐related mechanism). Another possibility is that proteins carrying these PTMs do not need to be imported into the ER from the cytosol, as there is increasing evidence that ubiquitin also has extracellular functions.^54^

**Figure 2:**
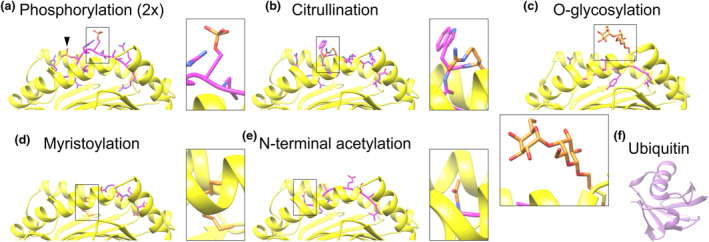
Examples of major histocompatibility complex (MHC) class I presentation of epitopes carrying post‐translational modifications of different sizes. Protein structures obtained by X‐ray diffraction of **(a)** MHC class I HLA‐B*27:05 carrying an epitope derived from SON phosphorylated at two positions [arrowhead and inset; Protein Data Bank (PDB) code: 7DYN].^116^ **(b)** HLA‐B*27:05 carrying a citrullinated epitope from VIPR (PDB: 3B6S).^56^ **(c)** Murine MHC class I H2‐K^b^ carrying an epitope from vesicular stomatitis Indiana virus nucleoprotein with an O‐linked glycan (2‐acetamido‐2‐deoxy‐beta‐d‐glucopyranose‐(1–4)‐[alpha‐l‐fucopyranose‐(1–6)]2‐acetamido‐2‐deoxy‐beta‐d‐glucopyranose) (PDB: 1KBG).^59^ **(d)** Rhesus macaque MHC class I molecule Mamu‐B*098 carrying a myristoylated epitope from the Simian immunodeficiency virus protein NEF (PDB: 4ZFZ).^60^ **(e)** HLA‐B*39:01 presenting an epitope from RNA helicase DDX3X with N‐terminal acetylation (PDB: 4O2C).^117^ **(f)** Ubiquitin, plotted at the same scale as the MHC peptide complexes of panels **a–e** (PDB: 4XOF).^118^ For **a–e**, post‐translational modifications (PTMs) are in orange.

Structural studies provide a solid understanding on how many post‐translationally modified peptides are presented by MHC class I and II and how this affects TCR interactions (Figure 2). PTMs can affect the binding of the peptide to MHC, the interactions of the peptide–MHC complex with the TCR, or both. Moreover, some MHC forms bind both the modified and the unmodified forms of the same peptide, as shown for phosphorylation^55^ citrullination^56^ and glycosylation.^19^ Although it might be expected that in these cases the PTM has no or only minor interactions with the peptide‐binding groove and the modified and nonmodified peptides thus bind in a similar conformation, this is not always the case. X‐ray crystallography of a phosphorylated epitope from melanoma antigen recognized by T cells 1 (MART‐1) in HLA‐DR1 revealed that, although the phosphate moiety was oriented away from the peptide‐binding groove, this modification altered the binding conformation of the N terminus of the epitope.^55^ Even more strikingly, it has been shown for an epitope from the self‐antigen vasoactive intestinal peptide type 1 receptor (VIPR) that the citrullinated peptide binds in a very different conformation to HLA‐B*27:05 and HLA‐B*27:09 compared with the nonmodified epitope.^56^

**Table 4:** Tyrosine modifications other than phosphorylation.

PTM	Protein	MHC‐I or ‐II	Allotype	Disease association	Reference
Nitration	Model antigen HEL	MHC‐II	I‐A^k^	Mouse model of infection	49
	Model antigen pigeon/moth cytochrome c	MHC‐II	I‐E^k^		50
	Birch (*Betula verrucosa*) pollen allergen Bet v 1.0101	MHC‐II	HLA‐DR	Allergy	98
Nitrosylation	Model antigen pigeon/moth cytochrome c	MHC‐II	I‐E^k^		50
Iodination	TG	MHC‐II	I‐A^k^, I‐A^s^, I‐E^k^ and HLA‐DR3	Experimental autoimmune thyroiditis	81, 82, 83, 84, 85, 86, 87, 88, 89, 90, 91, 92, 93, 94, 95, 96
*t*‐Butyl formation	BAX	MHC‐I	HLA‐A*02:01		108

HEL, hen egg lysozyme; MHC, major histocompatibility complex; PTM, post‐translational modification.

**Table 5:** Arginine modifications other than glycosylation.

PTM	Protein	MHC‐I or ‐II	Allotype	Disease association	Reference
Citrullination/deimination	Various	MHC‐II	HLA‐DR1	Rheumatoid arthritis	133
	ACAN	MHC‐II	HLA‐DR4		134
	FGA, VIME and ACAN	MHC‐II	HLA‐DRB1*04:01		65, 66
	VIME	MHC‐II	HLA‐DR4		135
	VIME	MHC‐II		Cancer	136
	VIME and MODI‐1	MHC‐II	HLA‐DR and DP4	Melanoma and ovarian cancer	39, 136, 137
	MMP21, CP1A2 and NMDE2	MHC‐II	HLA‐DP4	Melanoma	138
	ENO1	MHC‐II	HLA‐DR4	Pancreatic and lung cancer	139
	Hen egg white lysozyme (model antigen)	MHC‐II		Autophagy	140
	VIPR1	MHC‐I	HLA‐B27		56
	Various	MHC‐I		Cancer	15
	Guinea pig MBP	MHC‐II	RT1.B^L^		79
	ACAN and VIME	MHC‐II	HLA‐DRB1		67, 70
	HEL model antigen	MHC‐II	I‐A^k^		141
Methylation	Various	MHC‐I	HLA‐B*07:02	Leukemia	48
		MHC‐I			16
		MHC‐I		Cancer	15
Dimethylation	Various	MHC‐I			16
	HNRNPH3	MHC‐I	HLA‐B39		142
	Various	MHC‐I		Cancer	15
Hydrolysis to ornithine	Guinea pig MBP	MHC‐II	RT1.B^L^		79

HEL, hen egg lysozyme; MHC, major histocompatibility complex; PTM, post‐translational modification.

**Table 6:** Lysine modifications.

PTM	Protein	MHC‐I or ‐II	Allotype	Disease association	Reference
Hydroxylation	CII	MHC‐II	H‐2^q^	Collagen‐induced arthritis	32
Acetylation	Various	MHC‐I		Cancer	15, 17
	TP53	MHC‐II	HLA‐DR4 and DR53	Cancer	29
Methylation	Various	MHC‐I		Cancer	15, 17
Dimethylation	Various	MHC‐I		Cancer	15
N‐formylation	Various	MHC‐Ib	H2‐M3		143, 144, 145
Ubiquitination	Various	MHC‐I		Cancer	15
SUMOylation	Various	MHC‐I		Cancer	15
FAT10 linkage	Various	MHC‐I		Cancer	15
Homocitrullination/carbamylation	FLG	MHC‐II		Cancer	146
	VIME, ALDOA and KRT8	MHC‐II	HLA‐DR1 and ‐DR4		147
Trinitrophenyl conjugation	VSV nucleoprotein and BSA model antigens	MHC‐I	H‐2K^b^	Allergic contact dermatitis	101, 148, 149, 150
	Various	MHC‐I and ‐II			151
	Various	MHC‐II	I‐A^b^ and ‐A^d^		152

MHC, major histocompatibility complex; PTM, post‐translational modification; VSV, vesicular stomatitis virus.

**Table 7:** Other post‐translational modifications.

PTM	Protein	MHC‐I or ‐II	Allotype	Disease association	Reference
N(α)‐terminal acetylation	DBX1 and DDX3X	MHC‐I	HLA‐B39		117, 153
	MBP	MHC‐II	I‐A^u^	Experimental allergic encephalomyelitis	78, 154
	Various	MHC‐I		Cancer	15
Methylation (HNQILDE)	Various	MHC‐I		Cancer	15
Myristoylation	Nef	MHC‐I	Mamu‐B*098	Human and simian immunodeficiency viruses	60, 62, 63
	Synthetic epitopes	MHC‐I	HLA‐A*24:02 and ‐C*14:02		61
Spontaneous conversion of aspartic acid to isoaspartic acid	TRP2	MHC‐I	H‐2K^b^	Melanoma	155
Q deamidation	Various	MHC‐I		Cancer	15
	Wheat gliadin	MHC‐II	HLA‐DQ2 and DQ8	Celiac disease	71, 72, 73, 75, 76, 77, 156
	Various	MHC‐II	HLA‐DQ2 and DQ8	Type 1 diabetes	157
M oxidation	Various	MHC‐I		Cancer	15, 16
HP oxidation	Various	MHC‐I		Cancer	15
W oxidation	Model antigen HEL	MHC‐II	I‐A^k^	Infectious disease	15, 49
Proteasome protein splicing	FGF5	MHC‐I	HLA‐A3	Renal cell carcinoma	7
	PMEL	MHC‐I	HLA‐A32	Melanoma	8
	Various	MHC‐I		Leukemia and melanoma	17
Chemical ligation of non‐natural amino acid to fluorophore while presented on MHC	Model antigen OVA	MHC‐I	H2‐K^b^		113
	Influenza hemagglutinin	MHC‐II	HLA‐DR1		115

HEL, hen egg lysozyme; MHC, major histocompatibility complex; PTM, post‐translational modification.

In many cases, either the modified or unmodified form is preferentially presented by an MHC allotype, as best described for phosphorylation.^57^, ^58^ Moreover, these MHC allotypes present the PTM at specific sites of the epitope, which is not surprising because the MHC has to be able to accommodate the PTM.^15^, ^17^ For instance, phosphorylation of epitopes on MHC class I allotypes HLA‐B*40, ‐B*27, ‐B*39 and ‐B*07 is mostly at position 4, where the phosphate group can interact with a conserved arginine residue in the peptide‐binding groove of MHC.^23^, ^26^ However, this is not always the case. For instance, HLA‐B*40 also presents epitopes that are phosphorylated at P2, where the phosphoserine replaces the standard anchoring residue glutamic acid.^23^

As mentioned earlier, MHC‐presented peptides carrying large PTMs have been identified in peptide elution studies, and these modifications also occur at specific sites for different HLA forms. For example, ubiquitination was found to be enriched in position 3 for allotype HLA‐A*03:01.^15^ Although no structure of MHC presenting an ubiquitinated epitope is available, it is expected that the ubiquitin moiety is exposed toward the TCR, as its large size makes it difficult to fit in the peptide‐binding groove. Concerning MHC class I presentation of O‐GlcNAcylated peptides, HLA‐B*07:02 is a preferred allotype because it can accommodate the consensus sequence for O‐GlcNAcylation [P(V/A/T/S)g(S/T)], with the proline being the major anchor at position 2.^53^

Despite their size, large PTMs do not always obstruct TCR interactions. A crystal structure of a mouse MHC class I form with a O‐glycosylated epitope from vesicular stomatitis virus nucleoprotein showed that the glycan moiety is fully exposed and accessible for interactions with the TCR.^59^ Molecular modeling provided insights into how epitopes carrying such large glycosylation sites can be presented on MHC class II and still engage in TCR interactions.^18^ It was found that glycans are flexible, and that even large glycans can adopt conformations to allow for the simultaneous interaction of both the peptide–MHC complex and the glycan with the TCR.^18^

Finally, lipidations are another large PTM involving the conjugation of proteins to lipids. A crystal structure of an MHC class I form of the primate rhesus macaque carrying a myristoylated epitope showed that the fatty acid was inserted in a hydrophobic pocket of MHC and unlikely to interact with the TCR.^60^ A similar conformation was later reported for human MHC class I allotypes HLA‐A*24:02 and HLA‐C*14:02.^61^ Surprisingly, the peptides were only 4 or 5 amino acids long, which is shorter than for normal MHC class I presentation (8–11 residues), and most of these residues were also closely interacting with MHC, leaving only 2 or 3 amino acids available for interactions with the TCR.^60^, ^61^ In these cases, the shorter peptides were stably bound to MHC because the lipid moiety also contributed to the binding. This might be a general way of how lipidated peptides are presented by MHC because CTLs recognizing short myristoylated peptides (mostly 5 amino acids) have been identified in rhesus macaque.^62^, ^63^

## MHC PRESENTATION OF PTMs IN AUTOIMMUNITY AND ALLERGIES

The discovery of numerous T‐cell clones against post‐translationally modified epitopes indicates the importance of PTMs in the detection and clearance of infected and cancer cells. However, the aberrant recognition of post‐translationally modified proteins is also an important contributor to autoimmune diseases. This is best described in RA,^64^ which is hallmarked by T cells that respond to MHC class II carrying citrullinated epitopes derived from collagen II and other self‐antigens.^1^, ^32^, ^65^, ^66^, ^67^ Interestingly, in addition to citrullinated epitopes from collagen II, T cells against O‐glycosylated epitopes of the same autoantigen have also been described in RA,^34^ as reviewed elsewhere.^68^ Moreover, increased T‐cell responses to citrullinated MHC epitopes is not a general phenomenon, as T cells specifically recognizing both the citrullinated and nonmodified epitopes from influenza nucleoprotein have been found in infected mice.^69^

As specific HLA allotypes preferentially accommodate post‐translationally modified peptides, they are more prone to present certain PTMs than others. This is a key reason that certain HLA forms are associated with disease. For example, certain HLA‐DRB1 allomorphs are well known to present citrullinated epitopes derived from autoantigens vimentin and aggrecan because citrulline can be accommodated within the electropositive P4 pocket of HLA‐DRB1*04:01 and HLA‐DRB1*04:04, but not in the electronegative P4 pocket of the RA‐resistant HLA‐DRB1*04:02 form.^70^ As a result, these MHC class II allotypes are associated with RA and celiac disease.^70^ Similarly, HLA allotypes linked to celiac disease, such as DQ2 and DQ8, have a strong affinity for glutamine deamidated peptides derived from wheat gliadin.^71^, ^72^, ^73^, ^74^ These MHC class II molecules can accommodate peptides containing prolines and negatively charged amino acids. Although gluten peptides are rich in prolines, they typically contain fewer negatively charged amino acids. However, the deamidation introduces negatively charged glutamates, thereby increasing the binding affinity for DQ2 and DQ8.^71^, ^72^, ^73^, ^74^ T cells recognizing deamidated peptide from wheat gliadin have been identified in celiac disease.^71^, ^72^, ^73^, ^75^, ^76^, ^77^

In many cases, autoreactive T cells are well understood to contribute to disease progression. For example, an N‐terminally acetylated MHC class II epitope derived from myelin basic protein (MBP) can elicit experimental autoimmune encephalomyelitis, a mouse model of multiple sclerosis, whereas the nonacetylated form cannot.^78^ As with collagen II in RA, T cells specifically recognizing other post‐translationally modified epitopes from the same antigen have also been described, including glycosylated and citrullinated MHC class II epitopes.^79^ Finally, PTMs also play a role in allergies, as T cells recognizing only the glycosylated form of bee venom phospholipase A2 have been found in allergic human individuals.^80^

However, the best described example of incomplete tolerance for post‐translationally modified tissue‐restricted antigens is thyroglobulin. This protein is expressed in the thyroid gland and is essential for iodine uptake by facilitating the formation of iodinated tyrosine by thyroid peroxidase. Peptides derived from this protein carrying iodinated tyrosines can elicit potent T‐cell responses in an experimental murine model of autoimmune thyroiditis.^81^, ^82^, ^83^, ^84^, ^85^, ^86^, ^87^, ^88^, ^89^, ^90^, ^91^, ^92^, ^93^, ^94^, ^95^, ^96^ Consequently, the MHC presentation of this PTM has been proposed to be a key contribution in the pathogenesis of this autoimmune disease.^97^

In addition, non‐enzymatic peptide modifications, such a spontaneous oxidation induced by nitric oxide and reactive oxygen species during inflammation, have been linked to autoimmunity and allergies. One of these oxidation products is nitrotyrosine, which is formed from tyrosine under the influence of gaseous pollutants such as nitrogen oxides and ozone. It was found that nitrated major birch (*Betula verrucosa*) pollen allergen elicited a stronger T‐cell response in blood samples from patients with allergy compared with the nonmodified epitopes.^98^ Another non‐enzymatic PTM reported is cysteinylation, where a cysteine in the peptide forms a disulfide bond with a free cysteine molecule. This modification has been associated with graft‐*versus*‐host immune responses in organ rejection.^99^, ^100^ Finally, T cells can recognize MHC epitopes covalently modified by drugs, as it has been shown that trinitrophenyl conjugation could elicit specific cytolytic T‐cell responses in mice.^101^, ^102^

Some modifications can be both enzymatic and spontaneous. In a chimpanzee hepatitis C model, the enzymatic asparagine deamidation of a viral glycoprotein has been implicated in CD8^+^ T‐cell responses to this pathogen.^103^ However, asparagine can also be deamidated spontaneously: In a mouse study, it was found that the model antigen hen egg white lysozyme is nonspecifically deamidated and immunization with deamidated peptide can trigger specific CD8^+^ T‐cell responses.^104^

The presence of PTMs can not only promote, but also impair T‐cells responses, as some modifications prevent peptide binding to the MHC molecule and/or block the interaction with the TCR. For instance, T‐cell recognition of an MHC class II presented epitope derived from the autoantigen immunoglobulin kappa light chain was enhanced by reductive removal of the cysteinylation.^105^ This observation might explain why T‐cell responses to MHC presented on extracellular vesicles is diminished, as MS analyses revealed that MHC on extracellular vesicles presents higher levels of cysteinylated peptides compared with MHC presentation on cells.^16^ This increase in cysteinylation has been attributed to the longer presence of MHC–peptide complexes in extracellular vesicles, which increases the probability of non‐enzymatic modifications such as cysteinylation. However, cysteinylation might also occur before MHC presentation and extracellular vesicle formation, for instance, in the ER or in cysteine‐rich organelles.^99^

## THERAPEUTIC POTENTIAL OF PTMs

The potency of post‐translationally modified MHC presentation offers opportunities for drug development. For example, a bispecific antibody that binds to the TCR CD3 and a phosphopeptide presented in HLA‐A*02:01 was shown to target cytolytic T cells to cancer cells *in vitro*, resulting in T‐cell–induced apoptosis.^106^ Another therapeutic strategy for enhancing or lowering T‐cell responses is administering drugs that alter the prevalence of PTMs. For example, the compound ARS1620 covalently modifies the oncogene KRAS with the G12C mutation, and this can lead to drug‐modified MHC class I epitopes.^107^ These strategies can also be combined, as bispecific antibodies that cross‐link HLA‐A*02:01 presenting ARS1620‐modified epitope to CD3^+^ T cells could induce cancer cell killing even of cancer cells resistant to direct KRAS G12C inhibition.^107^

However, the high specificity of T‐cell responses against post‐translationally modified MHC epitopes can also lead to unwanted immune effects to protein‐modifying drugs. For example, in human blood samples, CD8^+^ T cells were identified that specifically recognize a synthetic epitope from the protein BAX with a *t*‐butyl modification.^108^ This modification does not normally occur in nature, but was a contamination in the synthetic peptide mixture. Although the mechanism by which CD8^+^ T cells develop specificity for this artificial modification remains unclear,^108^ it shows the potential risks of vaccination with artificial peptides and administration of protein‐modifying drugs.

An alternative approach for targeting MHC presentation of post‐translationally modified peptides is vaccination with peptides or proteins carrying a specific PTM, as has been best developed for phosphorylation. For example, aberrant phosphorylation of IRS2 and BCAR3 has been detected in a wide range of leukemias and solid tumors, including hepatocellular carcinoma, melanoma, ovarian cancer and colon cancer cell lines.^109^ Phosphorylated MHC epitopes from IRS2 and BCAR3 can activate both cultured human T cells and T cells *in vivo* in mouse cancer models.^109^ Initial clinical trials using these peptides for the treatment of high‐risk melanoma indicated that the peptides are safe while also resulting in specific T‐cell responses against the phosphorylated peptides.^110^

A similar strategy has also been tested for tolerogenic vaccination. Mouse studies showed that a fused peptide from collagen II with a phosphorylation site and fused to CLIP to overload MHC class II^111^ and a soluble MHC class II complex carrying glycosylated peptide^112^ can activate glycosylation‐specific T cells in a mouse model of collagen‐induced arthritis and thereby induce tolerogenic vaccination.

## CONCLUSION AND DISCUSSION

Post‐translational modifications play a crucial role in immune recognition by altering the peptide repertoire presented on MHC class I and II, influencing immune responses to both foreign pathogens and self‐antigens. While PTMs can help the immune system identify diseased cells, they can also contribute to autoimmunity and allergy.

Surprisingly, some PTMs, such as glycosylation and ubiquitination, are large modifications, raising questions about their processing, MHC loading and subsequent effects on T‐cell recognition. Several strategies can be considered for answering these questions. First, the proteolytic pathways for proteins carrying large PTMs can be studied using MS approaches and selective protease inhibitors. This will help clarify how large PTMs affect endo/lysosomal proteases needed for MHC class II presentation and the proteasome for MHC class I presentation. Second, it needs to be studied how interference in the enzymes responsible for generating or removing large PTMs, such as glycosyl transferases and (de)ubiquitinating enzymes, affects the MHC epitope repertoire. Third, functional T‐cell assays using synthetic peptides with and without PTMs can clarify how these modifications affect T‐cell recognition. Finally, structural analysis and modeling of MHC–peptide‐TCR complexes, as performed for glycosylation,^18^ can identify how MHC molecules accommodate large PTMs and whether T‐cell recognition is affected.

Another question is whether it is also possible that PTMs are generated on peptides already presented on MHC. Although it is technically challenging to demonstrate this, studies with peptides carrying non‐natural amino acids show that it is possible. For instance, MHC class I epitopes from the model antigen ovalbumin carrying non‐natural amino acids with bio‐orthogonal groups in surface‐accessible positions can be conjugated to fluorophores^113^ or chemically converted for improved T‐cell recognition^114^ while being presented on MHC. Similarly, it was shown using a similar bio‐orthogonal chemistry approach that synthetic peptides from influenza hemagglutinin can be fluorescently labeled while presented on the human MHC class II allotype HLA‐DR1.^115^

Thus, it is increasingly clear that the direct recognition of PTMs by T cells is crucial for identifying infected and malignant cells. Conversely, aberrant recognition of PTMs contributes to various autoimmune diseases and allergies. This understanding highlights the MHC presentation of PTMs as a promising target for developing novel therapies and vaccines to combat these pathologies.

## AUTHOR CONTRIBUTIONS

**Alexine S de Wit:** Writing – review and editing. **Frans Bianchi:** Writing – review and editing. **Geert van den Bogaart:** Writing – original draft; writing – review and editing.

## CONFLICT OF INTEREST

The authors declare no conflict of interest.
